# Adiponectin Receptors and Pro-inflammatory Cytokines Are Modulated in Common Variable Immunodeficiency Patients: Correlation With Ig Replacement Therapy

**DOI:** 10.3389/fimmu.2019.02812

**Published:** 2019-11-27

**Authors:** Rita Polito, Ersilia Nigro, Antonio Pecoraro, Maria Ludovica Monaco, Franco Perna, Alessandro Sanduzzi, Arturo Genovese, Giuseppe Spadaro, Aurora Daniele

**Affiliations:** ^1^Dipartimento di Scienze e Tecnologie Ambientali Biologiche Farmaceutiche, Università degli Studi della Campania “Luigi Vanvitelli,” Caserta, Italy; ^2^CEINGE-Biotecnologie Avanzate Scarl, Naples, Italy; ^3^Dipartimento di Scienze Mediche Traslazionali, Allergologia e Immunologia Clinica, Università degli Studi di Napoli Federico II, Naples, Italy; ^4^Dipartimento di Medicina Clinica e Chirurgia, Università degli Studi di Napoli “Federico II,” Naples, Italy

**Keywords:** adiponectin, common variable immunodeficiency, adiponectin receptors, leptin, cytokines

## Abstract

Adiponectin exerts beneficial pleiotropic effects through three receptors, AdipoR1, AdipoR2, and T-cadherin; it also exerts immunomodulatory effects. We previously demonstrated that adiponectin levels are altered in common variable immunodeficiency disease (CVID). The purpose of the present study was to investigate further the specific involvement of adiponectin in CVID by characterizing (i) the expression profile of adiponectin receptors on peripheral blood mononuclear cells; (ii) the levels of another relevant adipokine, namely leptin; (iii) the levels of five other cytokines (IL-2, IL-6, IL-10, TNFα, and IFNγ) in 24 patients on maintenance therapy, in 18 treatment-naïve patients (before and 24 h after the first Ig infusion) and in 28 healthy controls. We found that (i) adiponectin was down-expressed in patients on maintenance therapy and in treatment-naïve patients, and that it increased in treatment-naïve patients 24 h after the first Ig infusion; (ii) leptin expression did not differ between maintenance patients and controls either before or after the first Ig infusion; (iii) AdipoR1 expression was significantly higher on B lymphocytes, monocytes and NK cells of CVID patients than in controls; (iv) the expression of AdipoR1 and AdipoR2 on B lymphocytes, monocytes and NK cells was higher after the first Ig infusion than in treatment-naïve patients; (v) T-cadherin expression did not differ between treatment- naïve CVID patients and controls, and was not affected by Ig infusion; and (vi) IL-6, IL-8, IL-10, and TNFα levels were differently expressed in CVID patients on therapy maintenance and were not affected by the first Ig replacement therapy. This is the first study to demonstrate that the expression of AdipoRs in peripheral blood mononuclear cells from CVID patients differs from that of controls, and changes after the first Ig infusion. The specificity of adiponectin involvement in CVID is supported by the absence of changes in leptin levels and in the levels of the cytokines investigated. Taken together, these results suggest that the adiponectin system plays an important and specific role in CVID. A better understanding of adiponectin as a link in the cross-talk between the immune system and adipose tissue may provide additional benefits for the management of CVID patients.

## Introduction

Common variable immunodeficiency (CVID) comprises a group of heterogeneous disorders characterized by impaired antibody production (1, 2). It is the most common clinically symptomatic primary antibody disorder (prevalence: approximately 1:50.000 to 1:25.000) (3). CVID patients show also deregulation in the secretion of IL-2, IL- 4, IL-10, and IFN-γ by T cells (4). Adipose tissue is a source of adipokines involved in the pathogenesis and progression of metabolic and immune disorders and consequently plays a pivotal role in the control of metabolism and immunity (5). Adiponectin, that is produced by mature adipocytes, exerts beneficial effects on such cellular processes as energy metabolism, insulin sensitivity and inflammation (6). In particular, adiponectin levels are decreased in the metabolic diseases obesity (5, 6) and type 2-diabetes (5–7) but are elevated in classic chronic inflammatory/autoimmune diseases, such as asthma and chronic obstructive pulmonary disease (COPD) (8–10), multiple sclerosis and systemic lupus erythematosus (11, 12).

Adiponectin is a 244 amino acid monomer with a molecular weight of approximately 26 kDa. It is present in the circulation and accounts for up to 0.05% of total serum protein (13). It circulates as three oligomeric isoforms that differ in molecular weight: low molecular weight (LMW) trimers, medium molecular weight (MMW) hexamers and high molecular weight (HMW) multimers (13). The latter have been correlated with the most significant biological activities of adiponectin (13).

Adiponectin acts mainly through two receptors: AdipoR1 and AdipoR2 (14); a third non-signaling receptor has also been identified, T-cadherin (15, 16). AdipoRs are expressed in most tissues and cell lines including cells of the immune system, i.e., monocytes, B cells and NK cells, whereas they are barely expressed on T cells (17). AdipoR1 and AdipoR2 differ in both localization and binding affinity for adiponectin. Indeed, AdipoR1 is mainly expressed in skeletal muscle and binds globular adiponectin while AdipoR2 is mainly expressed in liver and engages the full-length adiponectin (14). Adiponectin negatively regulates lymphocyte functions (17). T-cadherin (also known as CDH13, cadherin 13, and H-cadherin) is abundantly expressed in injured vascular endothelial and smooth muscle cells in atherosclerotic regions (15). It is a receptor for the hexameric and high-molecular-weight species of adiponectin but not for the trimeric or globular species. Whether T-cadherin mediates signaling pathways is still controversial, but it is plausible that it serves as a reservoir of adiponectin (16).

Interest in the role of T-cadherin in human malignancies has recently increased consequent to the finding that it is down-regulated in several types of cancer (15) and that it regulates the progression of malignancies by modulating tumor cell proliferation and migration (16). Adiponectin has recently been found to be a modulator of the immune system that acts by inducing the secretion of the anti-inflammatory cytokines Interleukin (IL)-10 and Interleukin 1 Receptor Antagonist (IL-1RA), and by down-regulating the pro-inflammatory cytokines TNF-α and IL-6 (9, 18).

We recently demonstrated that adiponectin and in particular its HMW oligomers play an immunomodulatory role in CVID (19). In fact, we found that adiponectin levels are decreased, and correlated to IgA levels and associated with CVID phenotypes. In addition, adiponectin and HMW levels quickly and dramatically increased after the first Ig infusion in treatment-naïve CVID patients (19). In the attempt to shed further light on the role of adiponectin in CVID, we analyzed the expression profile of AdipoR1, AdipoR2 and T-cadherin on peripheral blood mononuclear cells (PBMC) from 18 treatment-naïve CVID patients, before and 24 h after the first Ig infusion. In addition, since cytokines are involved in the immunomodulation of CVID, we measured the serum expression of adiponectin, leptin, IL-2, IL-6, IL-10, TNF-α, and IFN-γ in 24 CVID maintenance patients, in 18 treatment-naïve CVID patients (before and after the first Ig infusion) and in 28 healthy controls.

## Materials and Methods

### Recruitment of Subjects

Twenty-four CVID patients on maintenance treatment with Ig (12 men, 12 women) and 18 (10 men and 8 women) treatment-naïve patients, diagnosed according to the European Society for Immunodeficiencies (2), were recruited by the Division of Allergy and Clinical Immunology of the Department of Translational Medical Sciences, University of Naples “Federico II.” As controls, we recruited 28 age-, body weight-, and body mass index-matched healthy volunteers from the staff of CEINGE-Biotecnologie Avanzate, Naples. Furthermore, T cell count and B cell subsets and the response to pneumococcal polysaccharide antigens were also measured data on serum Ig levels T cell count and B cell subsets at diagnosis, and clinical history were retrospectively retrieved from the medical files of CVID patients (19).

CVID maintenance patients received continuous Ig replacement therapy (0.4 g/kg/month) at intervals of 3 weeks to maintain Ig levels above 600 mg/dl (768 ± 87 mg/dl). There were no familial cases of CVID in the control group. Treatment-naïve patients received intravenous Ig immunomodulating therapy at a dose of 0.4 g/kg. The research protocol was approved by the Ethics Committee of the School of Medicine, University of Naples “Federico II” and was conducted in accordance with the principles of the Helsinki II Declaration. Written informed consent was obtained from all participants.

### Anthropometric and Biochemical Investigations

The height and weight of the patients were measured using standard techniques and the body mass index was calculated as body weight (kg)/height^2^ (m^2^). Blood samples (5 ml) were taken after 12 h of fasting from maintenance patients, and from treatment-naïve CVID patients before the first Ig replacement therapy (0.4 g/kg) and 24 h after. Serum samples were immediately centrifuged and aliquots were stored at −20°C. The levels of IgG, IgA, IgM, total cholesterol, HDL, and LDL cholesterol, triglycerides, glucose, total proteins, iron, fibrinogen, C reactive protein (CRP) and erythrocyte sedimentation rate (ESR) were determined in all patients with standard enzymatic methods (Hitachi Modular, Roche, Mannheim, Germany).

### Measurement of Adiponectin and Leptin

Serum concentrations of adiponectin and leptin were evaluated in 18 naïve CVID patients before and 24 h after the first Ig infusion, in the 24 CVID patients on maintenance therapy and in the 28 healthy controls. The concentration of total adiponectin was measured by enzyme-linked immunosorbent assay (ELISA) as previously reported (20). Leptin levels were measured using an ELISA commercial kit (Elabscience, Houston, Texas, USA).

### Assessment of Adiponectin Receptor Expression on Peripheral Blood Mononuclear Cells by Flow Cytometry

Leukocyte adiponectin receptor expression (AdipoR1, AdipoR2, and T cadherin) was evaluated in 18 treatment-naïve CVID patients before and after the first administration of Ig. PBMC were stained with the specific antibodies for 30 min at 4°C. Subsequently, samples were labeled with the relevant secondary conjugated antibodies for 30 min at 4°C. Isotype controls and secondary only conditions served as negative controls. Rabbit anti-human AdipoR1 (357–375) and AdipoR2 (374–386) antibodies (Phoenix Pharmaceuticals, Karlsruhe, Germany) were used at 5 μg/ml and detected using 8 μg/ml goat-anti rabbit Alexa 488 secondary antibody (Life Technologies, Milan, Italy). Gating to measure the expression of AdipoR1 and AdipoR2 on PBMC and B cells was based on the isotype control. Isotype control frequencies were subtracted from the AdipoR1 and AdipoR2 frequencies in each subject. The following antibodies were used to stain human PBMC: CD4-FITC (1:50) (OKT-4), CD3-PerCp-Cy5.5 (1:50) (OKT3), CD19-PECy7 (1:50) (HIB19), CD8-Pacific Blue (1:50) (OKT8), CD56-PE (1:50) (MEM188) (all from E-bioscience, Hatfield, UK), CD4-Pacific orange (1:10) (clone S3.5) (Life Technologies, Milan, Italy) and CD45RO-APC (1:20) (UCHL1) (BD Bioscience, Oxford, UK), α4β1-PE (1:100) (P5D2), αLβ2-FITC (1:100) (212701), DP-2-FITC (1:10) (301108) (R&D Systems, Abingdon, UK.), CXCR3-PE (1:50) (2Ar1) (VWR International PBI S.r.l., Milano, Italy). B cell subsets were labeled using CD19-PerCp-Vio700 (1:30) (LT19), IgM-PE (1:30) (PJ2-22H3), IgD-APC (1:60) (IgD26), CD38-FTIC (1:150) (IB6), and CD27-APC-Vio-770 (1:10) (M-T271) (all from Miltenyi Biotec, Bergisch Gladbach, Germany).

### Measurement of Cytokine Levels

The levels of 8 cytokine species (IL-2, IL-4, IL-6, IL-8, IL-10. INFγ, TNFα, GM-CSF) were measured in the serum of the 18 treatment-naïve CVID patients before and 24 h after the first Ig infusion, in the 24 maintenance therapy CVID patients and in 28 healthy controls using a commercially available kit (Bio-Plex Pro™ Human Cytokine 8-plex Assay, Hercules, CA, USA). The assay was performed according to the manufacturer's instructions and the concentrations of cytokines were calculated by comparing reads with a 5-parameter logistic standard curve using a Bioplex-200 instrument (Bio-Rad, Hercules, CA, USA).

### Statistical Analysis

Statistical significance was established at *p* < 0.05. Bonferroni and Student's *t*-tests were used to compare the means of biochemical parameters. Analysis of variance (ANOVA) and the Bonferroni *t*-test were used to compare mean cytokine levels.

## Results

### Anthropometric and Biochemical Features of CVID Patients

The anthropometric and biochemical characteristics of the 24 CVID patients on maintenance therapy, and the 28 healthy controls are reported in Table 1. The levels of total cholesterol, total proteins and iron were lower in patients than in controls (*p* < 0.03). The results of the ELISA test confirmed the lower total adiponectin levels in CVID patients vs. control subjects (*p* = 0.03), and moreover show that total adiponectin levels increased in treatment-naïve patients 24 h after the first Ig replacement treatment (*p* = 0.007). Table 2 shows the characteristics of the 18 treatment-naïve CVID patients before and 24 h after the first Ig replacement.

**Table 1:** Anthropometric and biochemical features of CIVD patients on maintenance therapy, and in treatment-naïve patients and controls.

	**Controls**	**Maintenance therapy patients**	***p-value***	**Naive patients** **n. 18**	***p-value***
Sex M/F	14/14	12/12		10/8	
Age (years)	42.67 (15)	45.29 (14.86)	0.53	41.44 (16.18)	0.42
Body Mass Index (kg/m^2^)	24.36 (2.74)	25.19 (4.45)	0.45	24.51 (4.47)	0.65
Total Cholesterol (mg/dl)	199.88 (43.17)	161.41 (41.21)	**0.002**	158.37 (28.33)	0.87
Triglycerides (mg/dl)	95.56 (45.25)	95.16 (35.80)	0.97	94.66 (56.52)	0.97
Glycemia (mg/dl)	87.29 (13.86)	80.29 (15.31)	0.09	82.06 (10.79)	0.69
IgG (mg/dl)	–	224.79 (97.56)	–	2.36 (1.85)	**5.47**^**−12**^
IgA (mg/dl)	–	12.41 (13.99)	–	0.11 (0.13)	**0.0006**
IgM (mg/dl)	–	23.75 (47.88)	–	0.31 (0.38)	0.07
Total proteins (mg/dl)	7.3 (0.62)	6.48 (0.59)	**0.001**	6.01 (0.54)	
Iron (μg/dl)	95.59 (34)	68.79 (34.12)	**0.01**	55.75 (26.04)	0.20
Fibrinogen (mg/dl)	–	331.54 (99.66)	–	301.81 (66.44)	0.30
C reactive protein (mg/dl)	–	0.678 (0.78)	–	2.21 (2.87)	**0.02**
ESR (mm)	–	10.16 (9.75)	–	8.4 (4.83)	0.59
Adiponectin (μg/ml)	20.17 (8.74)	15.96 (3.63)	**0.03**	6.53 (6.19)	**2.55**^**−07**^
Leptin (ng/ml)	9.49 (3.59)	8.38 (3.75)	0.28	8.32 (3.29)	0.95

*Data are expressed as mean (sd)*.

*Statistically relevant values are reported in bold*.

**Table 2:** Anthropometric and biochemical features of treatment-naïve CVID patients before and 24 h after the first Ig infusion.

	**Naïve patients** **n.18**	**Naïve patients—24 h post Ig infusion**	***p-value***
Sex M/F	10/8	–	–
Age (years)	41.44 (16.18)	–	–
Body Mass Index (kg/m^2^)	24.51 (4.47)	–	–
Total Cholesterol (mg/dl)	158.37 (28.33)	–	–
Triglycerides (mg/dl)	94.66 (56.52)	–	–
Glycemia (mg/dl)	82.06 (10.79)	–	–
IgG (mg/dl)	2.36 (1.85)	7.99 (2.83)	**0.54**^**−8**^
IgA (mg/dl)	0.11 (0.13)	0.12 (0.15)	0.78
IgM (mg/dl)	0.31 (0.38)	0.33 (0.35)	0.87
Total proteins (g/dl)	6.01 (0.54)	–	–
Iron (μg/dl)	55.75 (26.04)	–	–
Fibrinogen (mg/dl)	301.81 (66.44)	–	–
C reactive protein (mg/dl)	2.21 (2.87)	–	–
ESR (mm)	8.4 (4.83)	–	–
Adiponectin (μg/ml)	6.53 (6.19)	13.93 (9.22)	**0.007**
Leptin (ng/ml)	8.32 (3.29)	9.01 (4.88)	0.62

*Data are expressed as mean (sd)*.

*Statistically relevant values are reported in bold*.

### Leptin Concentrations in CVID Patients

To verify the specificity of adiponectin modulation in CVID, we investigated the involvement of leptin, which is one of the most pivotal cytokines produced by adipose tissue. First, to explore whether the levels of leptin are modulated by Ig replacement therapy, we measured their levels in treatment-naïve CVID patients before and 24 h after the first Ig replacement therapy. Interestingly, unlike adiponectin, leptin levels were not affected by Ig infusion (Table 2). Accordingly, leptin concentrations did not differ between CVID patients on maintenance therapy and controls (Table 1).

**Figure 1:**
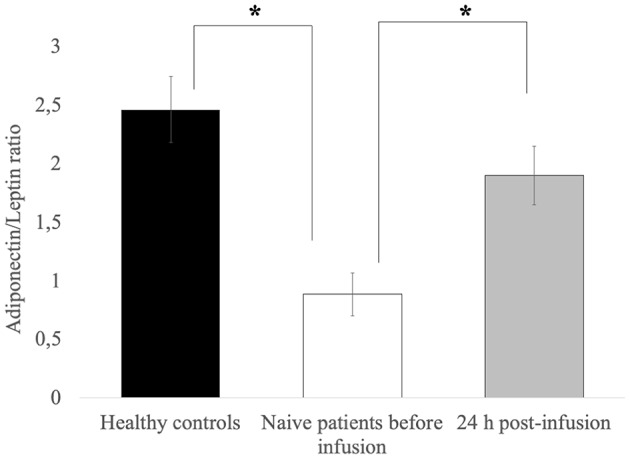
Leptin levels are comparable in controls and patients, and are unchanged after the first Ig infusion. The adiponectin-leptin ratio in patients on maintenance therapy and in 16 treatment-naïve CVID patients before and 24 h after the first Ig infusion. Data represent the mean (±standard deviation of three independent experiments, each performed in triplicate. *p ≤ 0.05.

Figure 1 shows adiponectin and leptin levels and the adiponectin/leptin ratio in treatment-naïve CVID patients before and 24 h after the first Ig replacement therapy vs. healthy controls. The adiponectin-leptin ratio was consistently lower in treatment-naïve patients than in controls. On the contrary, the adiponectin-leptin ratio increased 24 h after the first Ig replacement infusion (Figure 1). Given that Ig infusion did not affect leptin levels, we conclude that this difference in ratio values is attributable to variations in adiponectin expression.

### AdipoR1, AdipoR2, and T-Cadherin Expression on PBMC

**Figure 2:**
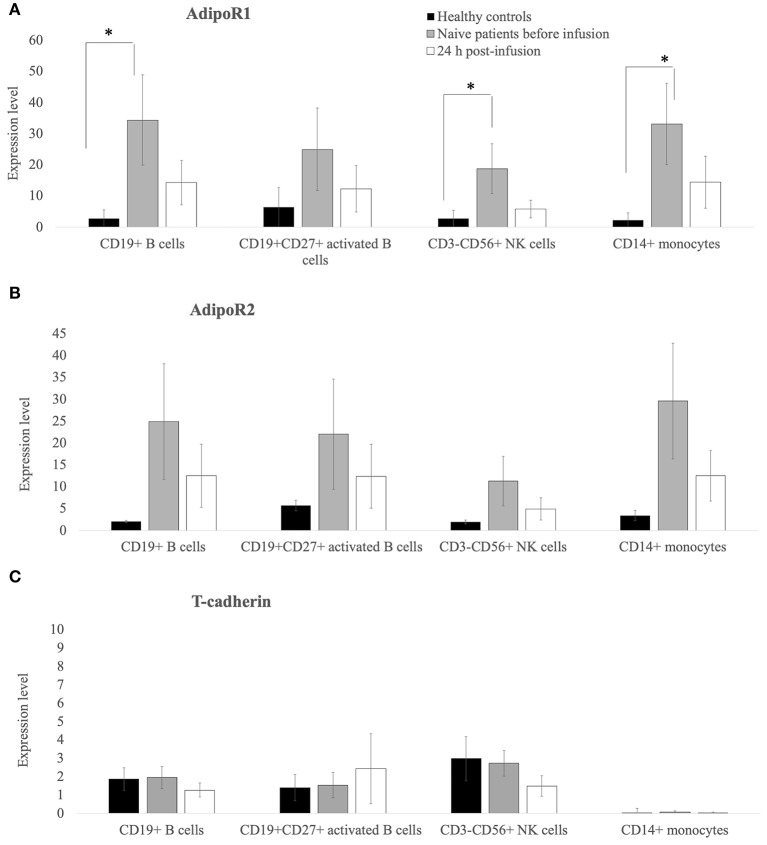
AdipoR1 and AdipoR2 expression was higher in lymphocyte subpopulations of treatment-naïve CVID patients than in those of healthy controls. Their expression decreases 24 h post the first Ig replacement therapy. **(A–C)** Percentage of AdipoR1- AdipoR2- and T-cadherin-positive cells on the lymphocyte subpopulations (CD19+ B cells, CD19+CD27+ B-activated cells, CD3–CD56+ NK lymphocytes and CD14+ monocytes) from healthy controls and treatment-naïve CVID patients before and 24 h after the first Ig infusion. Data obtained from two independent experiments performed by flow-cytometry in triplicate. *p ≤ 0.05.

As shown in Figure 2, flow cytometry demonstrated that the expression (in terms of the percentage of positive cells) of AdipoR1 and AdipoR2 on the surface of CD19+ B cells, CD19+CD27+ activated B cells, CD3-CD56+ NK cells, and CD14+ monocytes (Figures 2A,B) was higher in treatment-naïve CVID patients than in healthy controls. Notably, AdipoR1 expression on CD19+ B cells, CD3– CD56+NK cells and CD14+ monocytes in CVID patients was significantly higher than in healthy controls whereas AdipR1 expression on CD27+ B cells did not differ significantly from controls (Figure 2A). AdipoR2 expression on CD19+ B cells, CD– CD56+NK, CD14+ monocytes and CD27+ B cells was higher in CVID treatment-naïve patients than in controls although the difference was not significant (Figure 2B).

Interestingly, 24 h after the first Ig replacement therapy, the levels of both AdipoR1 and 2 decreased on the surface of CD19+B cells, CD19+CD27+activated B cells, CD3–CD56+ NK cells, and CD14+ monocytes. The expression of T-cadherin on activated B cells, NK or monocytes did not differ between patients and controls after the first Ig replacement therapy (Figure 2C). We also collected blood samples from 5 patients and analyzed AdipoRs expression in PBMC at 7, 14, and 21 days post-infusion and found that the data did not differ among the various time points examined (Supplementary Figure 1). The expression of AdipoR1, 2, and T-cadherin on T-lymphocytes was barely detectable (data not shown).

### The Cytokine Profile in Maintenance Therapy CVID Patients

**Figure 3:**
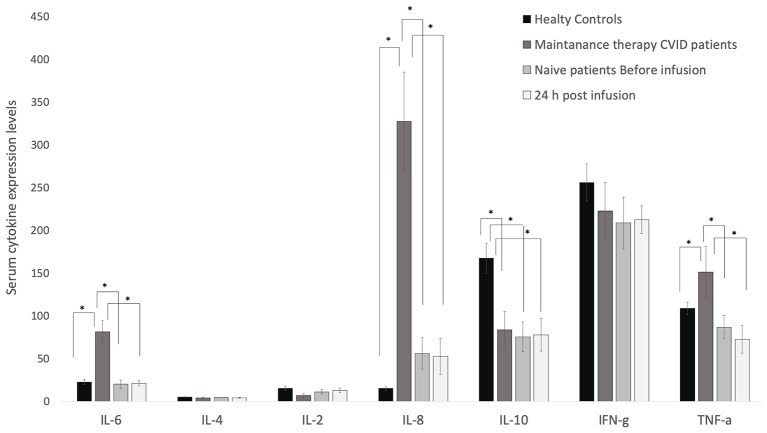
Serum levels of IL-6, IL-8 and TNF-α were higher in CVID patients than in healthy controls. Their levels are barely modified by the replacement therapy. ELISA assay was performed to quantify serum levels of cytokines in CVID patients and healthy controls. IL-6, IL-4, IL-2, IL-8, IL-10, INFγ, and TNF-α were quantified in sera from healthy controls, maintenance CVID patients, and treatment-naïve patients before and 24 h after the first Ig replacement therapy. Data obtained from two independent experiments performed in triplicate *p ≤ 0.05.

As shown in Figure 3, the levels of IL-6, IL-8, and TNF-α were significantly higher in the two treatment groups than in controls, and IL-10 levels were significantly lower in patients than in controls, while the expression of IL-2, IL-4, and INF-γ did not differ significantly between the two groups of patients (maintenance and naïve) and controls. The levels of IL-6, IL-8, and TNF-α in treatment naïve patients were comparable to those of healthy controls. The first infusion of Ig barely modified cytokine levels after 24 h or 7, 14, and 21 days later (Supplementary Figure 2). IL-10 serum levels were significantly lower in naïve patients than in controls (Figure 3).

## Discussion

We recently reported that the expression of total adiponectin and its HMW oligomers is decreased in patients with CVID, that it is associated with CVID activity and that it is correlated to the first Ig infusion (19). Here we demonstrate that the expression of AdipoR1 and AdipoR2 in PBMCs from CVID patients differs from that of controls, and changes after the first Ig infusion. The specificity of adiponectin involvement in CVID is supported by the absence of changes in leptin expression and in the levels of various cytokines tested. Taken together these results suggest that the adiponectin system plays a role in CVID.

The expression of AdipoR1 and AdipoR2 has been studied on B lymphocytes, monocytes, and NK cells but not on T lymphocytes (21). AdipoR1 and AdipoR2 expression was found to be decreased on the surface of B lymphocytes of patients with autoimmune disorders, namely, rheumatoid arthritis and type 1 diabetes (22). Chimen et al. reported an inverse association between adiponectin serum levels and AdipoR expression on immune cells (23). Accordingly, in an earlier study we found that low levels of adiponectin in CVID are accompanied by up-regulation of AdipoR1 and AdipoR2 and, *vice versa*, that high serum levels of adiponectin in autoimmune disorders result in the down-regulation of AdipoR1 and AdipoR2 (5). In addition, our findings suggest that the immune regulation functions of adiponectin in CVID are specifically related to AdipoR1 and 2 but not to T-cadherin signaling.

While T-cadherin expression has been associated with endothelial injuries and cancer progression (24, 25), to our knowledge, there are no data about the expression of T-cadherin on immune cells. The absence of changes in T-cadherin expression on the PBMC of our patients suggests that, in CVID, this receptor is not involved in the immune functions of adiponectin.

Another interesting result of our study concerns the changes in AdipoR1 and AdipoR2 expression, which, similar to the changes in adiponectin levels, were partially restored to “normal” levels after the first Ig treatment. This novel finding strengthens the concept that the adiponectin system plays a pre-eminent role in CVID. To assess the specificity of adiponectin involvement in CVID we looked at leptin. The latter cytokine is produced by adipose tissue, and is reported to play a key role in immunity (26, 27). We found that leptin expression did not differ between maintenance patients and controls, nor before and after the first Ig treatment in treatment naïve CVID patients. In the only study conducted thus far on leptin in CVID, leptin expression was not associated to the disease (28).

Various studies have been devoted to the production of cytokines in CVID, albeit with conflicting results (4, 29, 30). In the present study we found (i) that IL-6, IL-8 and TNF-α levels were higher, (ii) that L-10 levels were lower, and (iii) that IL-2, IL-4, and INF-γ were not differently expressed in patients vs. controls. Moreover, we found that in treatment-naïve patients, the levels of most cytokines were comparable to those of healthy controls with the exception of IL-10, the levels of which were significantly lower in treatment-naïve patients than in controls (31, 32). The different behavior between cytokine levels in maintenance vs. absence of modifications in treatment-naïve patients is probably related to the patient's condition (i.e., infections, cancer, etc.). Indeed, changes in the cytokine expression are evident only after several Ig administrations, as observed in patients on maintenance therapy. IL-6 and IL-8 are mainly produced and secreted by macrophages thereby enhancing the proliferation and the differentiation of B cells into memory or plasma cells (33, 34), while IL-2 and IL-4 are produced in most part by T lymphocytes (35). The up-regulation of IL-6 and IL-8 in CVID maintenance therapy may indicate a primary involvement of macrophages instead of T lymphocytes. We believe that the continuous infusion of Ig might activate Th2 and macrophages, thereby resulting in the release of IL-6 and IL-8. Elevation of IL- 6 and IL-8 was reported in CVID patients by Varzaneh et al. (4) and by Ibanez et al. (36). In addition, persistent activation of macrophages, which are the major sources of IL6 and IL8, was seen in CVID patients on maintenance therapy (37).

IL-10 serum levels are closely related to those of IL-2 and IL-4, which are low in naïve patients due to the alteration of the immune system typical of CVID patients (38). In fact, impaired secretion of IL-10 by the T-cells of CVID patients has been widely reported (32, 39, 40).

In conclusion, this is the first study to demonstrate that adiponectin receptors are differentially expressed on PBMC from CVID patients and that their expression is partially restored after the first Ig infusion. The peculiarity and relevance of the role played by adiponectin in CVID is confirmed by the finding that leptin and the other cytokines tested herein did not change after the first Ig infusion in treatment-naïve CVID patients. Further studies are needed to better understand the molecular mechanisms underlying the effects exerted by adiponectin in the pathogenesis of CVID.

## Data Availability Statement

All datasets generated for this study are included in the article/Supplementary Material.

## Ethics Statement

The studies involving human participants were reviewed and approved by Ethics Committee of the School of Medicine, University of Naples Federico II. The patients/participants provided their written informed consent to participate in this study.

## Author Contributions

RP, EN, and MM performed the experiments. RP, AD, and EN wrote the manuscript. AP, AG, and GS recruited the patients and samples. FP and AS performed flow-cytometry experiments. GS and AD conceived the study and revised and approved the final version of the manuscript.

### Conflict of Interest

The authors declare that the research was conducted in the absence of any commercial or financial relationships that could be construed as a potential conflict of interest.
