# Supplementary material for: Adiponectin Receptors and Pro-inflammatory Cytokines Are Modulated in Common Variable Immunodeficiency Patients: Correlation With Ig Replacement Therapy
Source: Front Immunol. 2019 Nov 27;10:2812. doi: 10.3389/fimmu.2019.02812 (PMC6890605; doi:10.3389/fimmu.2019.02812)
Supplement: Supplementary file 1 [file Presentation_1.pdf]

Supplementary figure 1: AdipoR1 and AdipoR2 expression in lymphocyte subpopulations of treatment-naïve CVID patients than in those of healthy controls. AdipoR1- and AdipoR2- positive cells on the lymphocyte subpopulations (CD19+ B cells, CD19+CD27+ B-activated cells, CD3-CD56+ NK lymphocytes and CD14+ monocytes) from healthy controls and treatment-naïve CVID patients before and 24 hours, 7 days, 14 and 21 days after the first Ig infusion. Data obtained from two independent experiments performed by flow-cytometry in triplicate. *$p \leq 0.05$.

Supplementary figure 2: Serum levels of IL-6, IL-4, IL-2, IL-8, IL-10, INFγ and TNF-α in CVID patients and in healthy controls. ELISA assay was performed to quantify serum levels of cytokines in healthy controls, maintenance CVID patients, and treatment-naïve patients before and 24 hours, 7 days, 14 and 21 days after the first Ig replacement therapy. Data obtained from two independent experiments performed in triplicate. *$p \leq 0.05$.
