# Supplementary figures and images for: Adiponectin Receptors and Pro-inflammatory Cytokines Are Modulated in Common Variable Immunodeficiency Patients: Correlation With Ig Replacement Therapy
Source: Front Immunol. 2019 Nov 27;10:2812. doi: 10.3389/fimmu.2019.02812 (PMC6890605; doi:10.3389/fimmu.2019.02812)

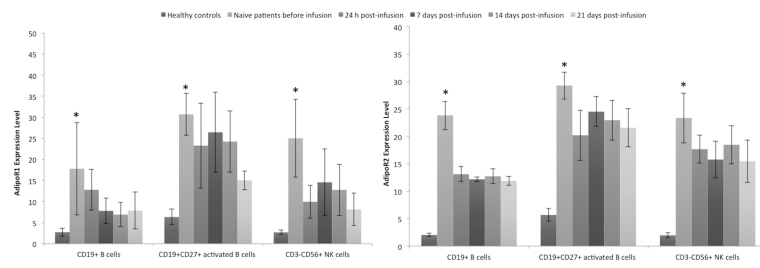

Supplement: Supplementary file 2 [file Image_1.pdf]

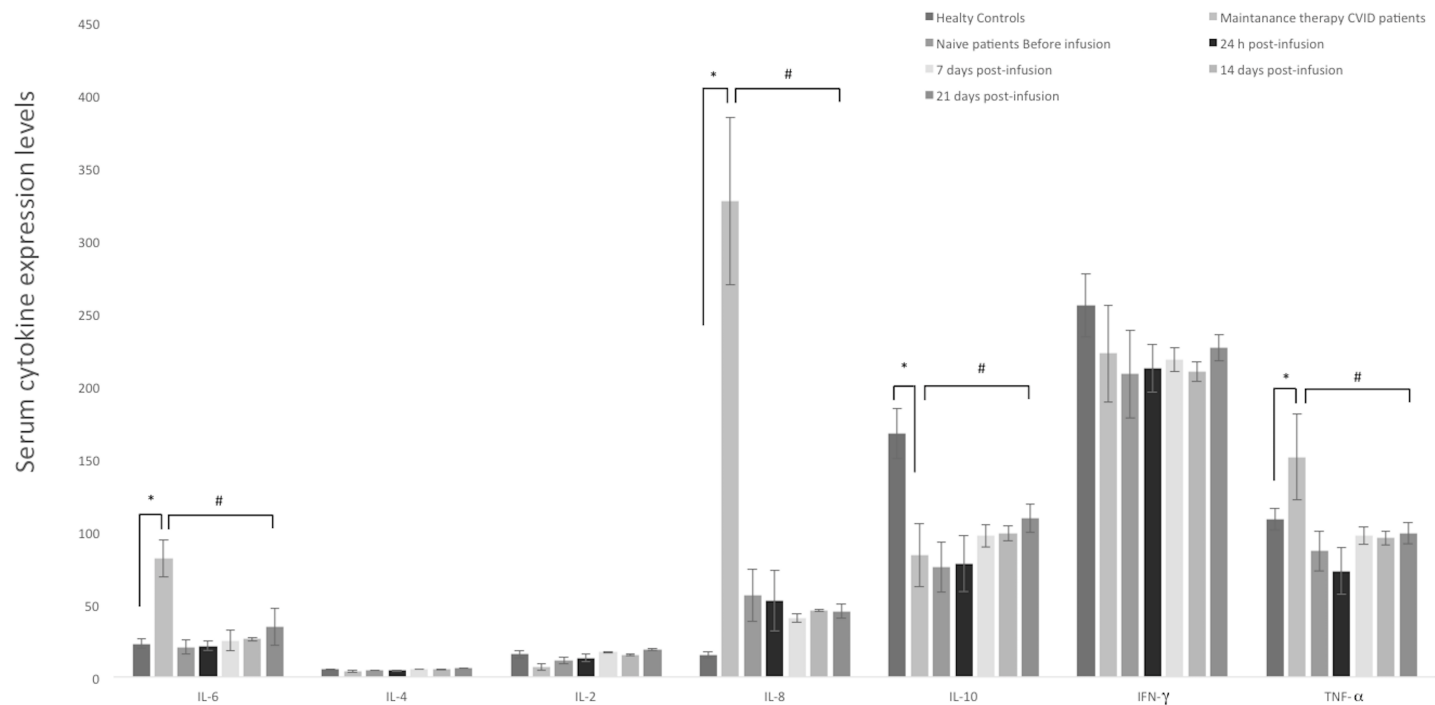

Supplement: Supplementary file 3 [file Image_2.pdf]
